# Study on the construction and application effect of discharge preparation services in heart valve replacement surgery

**DOI:** 10.3389/fcvm.2026.1879911

**Published:** 2026-10-30

**Authors:** Chunyan Zhang, Huan Yu, Wei Zhai, Lili Zhang, Ting Zhang, Yuanyuan Li

**Affiliations:** Department of Cardiovascular Surgery, The Second Hospital of Tianjin Medical University, Tianjin, China

**Keywords:** application effect, discharge preparation service, heart valve disease, heart valve replacement surgery, unplanned readmission

## Abstract

**Objective:**

To develop and evaluate a discharge preparation service (DPS) for patients after heart valve replacement surgery.

**Method:**

This retrospective cohort study included 323 patients who underwent heart valve replacement surgery between February 2023 and January 2025. A multidisciplinary DPS plan was developed using a modified Delphi method and implemented in clinical practice. After 1:1 propensity score matching, 110 patients receiving DPS and 110 receiving usual care were analyzed. RHDS and QDTS were assessed at discharge; CSMS, CCQQ, and NSNS were assessed at discharge and at 3 and 6 months after discharge. Linear mixed models examined time, group, and time × group interactions.

**Results:**

All items in the DPS plan were agreed upon by experts (average importance score ≥4.0, coefficient of variation ≤0.25), with a Kendall's coefficient of concordance W value of 0.925 (*P* < 0.001), and the expert authority coefficient was 0.914. After propensity score matching (PSM), there were no significant differences in baseline characteristics between the two groups (*P* > 0.05, standardized mean difference <0.1). Compared with controls, the DPS group had significantly higher RHDS and QDTS scores at discharge (*P* < 0.05). For CSMS, CCQQ, and NSNS, time × group interactions were significant [CSMS: F(2,436) = 77.73; CCQQ: F(2,436) = 93.92; NSNS: F(2,436) = 67.53; all *P* < 0.001], indicating steeper improvement trajectories with DPS. At 6 months after discharge, the interaction estimates were 11.23 (95% CI 9.23–13.23) for CSMS, 15.61 (95% CI 13.36–17.86) for CCQQ, and 10.40 (95% CI 8.60–12.20) for NSNS (all *P* < 0.001). DPS was associated with lower unplanned readmission (6.36% vs. 15.45%, *P* = 0.031) and lower total complications (25.45% vs. 59.09%, *P* < 0.001).

**Conclusion:**

The systematic, multidisciplinary DPS plan was associated with significant improvements in discharge readiness, self-management ability, and quality of life, as well as lower rates of unplanned readmissions and complications after heart valve replacement surgery.

## Introduction

Valvular heart disease is one of the fastest-growing cardiovascular diseases worldwide, and its incidence is closely related to the process of population aging (1). Epidemiological data show that among people under the age of 65, the incidence rate is usually below 2%; while in the elderly population over 75 years old, this rate sharply increases to 13.2% (2). This trend indicates that as the degree of social aging continues to increase, heart valve diseases have increasingly become a significant disease affecting public health and burdening the medical system (3). According to a survey conducted among the Chinese population, it is estimated that there are currently approximately 25 million patients with valvular heart disease in China, and the overall prevalence rate has reached 3.8% (4). For a long time, the main treatment method for severe heart valve diseases has been traditional open-chest surgery. However, this method has significant drawbacks such as large trauma and long recovery period. Moreover, it significantly increases the surgical risks for elderly patients, those with multiple systemic diseases, or those with poor cardiac function, thereby limiting the treatment options for some patients (5).

In recent years, with the rapid development of cardiology technology, heart valve replacement has gradually matured and been widely applied in clinical practice, providing patients with safe and effective surgical solutions (6–8). Compared with traditional surgical procedures, it has the advantages of less trauma, faster recovery and shorter hospital stay, enabling more elderly and high-risk patients to have the opportunity for treatment (9). However, this technology did not alter the underlying pathological basis of the patient's own valves or the overall cardiovascular risks associated with them. After the surgery, the patient still faced multiple risks such as valve dysfunction, heart failure, arrhythmia, bleeding and thrombosis events (10). In addition, due to the rapid transition during the recovery period, patients often encounter challenges such as insufficient self-management knowledge, complex medication regimens, and poor compliance with rehabilitation behaviors, resulting in a persistently high rate of unplanned readmission, which significantly affects long-term prognosis and quality of life (11). Therefore, how to effectively assist patients in achieving a safe and smooth transition from inpatient treatment to home-based rehabilitation has become a crucial aspect for improving the long-term prognosis after heart valve replacement surgery.

The Discharge Preparation Service (DPS) is a patient-centered, structured and systematic intervention model (12). Unlike routine discharge education, which is typically a single pre-discharge session focusing on medication, activity, diet, and follow-up instructions, DPS is a multi-stage, multidisciplinary pathway from admission to 6 months after discharge, integrating needs assessment, individualized goal setting, health education, medication management, cardiac rehabilitation, nutritional and psychological support, emergency guidance, and continuous follow-up. It also differs from general transitional care, which mainly focuses on post-discharge care coordination, by embedding structured discharge preparation within a standardized multidisciplinary pathway (13). The transition theory provides an important theoretical framework for such interventions, emphasizing the necessity of providing personalized support during the role and environment transition process (14). In recent years, domestic studies have begun to explore discharge preparation service plans for postoperative patients, confirming their positive effects in improving discharge readiness and short-term prognosis (15). Although discharge preparation and transitional care can improve discharge readiness and short-term outcomes, evidence on mid-term trajectories of self-management ability, quality of life, and nursing satisfaction after a standardized, multidisciplinary DPS pathway remains limited, and few studies have examined time × group interactions or the impact of structured DPS on unplanned readmission and complications after heart valve replacement surgery.

Therefore, this study aims to implement a systematic, multi-stage, evidence-based DPS plan, and adopt a retrospective cohort study method to explore the comprehensive impact of this plan on the rehabilitation readiness status of patients after heart valve replacement surgery at discharge and the mid-term rehabilitation effect after discharge. The primary outcomes were discharge readiness (RHDS) and discharge guidance quality (QDTS) at discharge, and self-management ability (CSMS), quality of life (CCQQ), and nursing satisfaction (NSNS) at discharge and at 3 and 6 months after discharge. Secondary outcomes included unplanned readmission and complications within 6 months. This is in the hope of providing more solid clinical practice evidence for optimizing the whole-process management of patients after heart valve replacement surgery and promoting the effective DPS model.

## Materials and methods

### The delphi study of the DPS plan

The team-based systematic DPS scheme for patients after heart valve replacement was developed through the modified Delphi method (16). The study began with the establishment of a multidisciplinary team that included cardiac surgeons, cardiac specialist nurses, and clinical pharmacists. The team performed a systematic review of existing literature and successfully synthesized 31 pieces of top-tier evidence (13, 17). The aim is to identify the key challenges in the transitional care during the period from hospital admission to discharge for patients after heart valve replacement surgery, the core elements of the multidisciplinary team (MDT) collaboration framework, and their potential applications in accelerated rehabilitation surgery and chronic disease (anticoagulation treatment) management. A preliminary systematic DPS plan covering admission to 6 months after discharge was drafted in five stages and formed the basis for the first Delphi round. Sixteen experts were selected from six key areas: cardiac surgery, nursing, clinical pharmacy, rehabilitation medicine, nutrition, and psychology. The selection criteria were: (1) senior title or higher; (2) no less than ten years of experience in perioperative care of cardiac surgery, cardiac rehabilitation, or chronic disease (especially anticoagulation treatment) management; (3) familiarity with patient education, continuous care, or interdisciplinary team collaboration. The expert panel consisted of 16 experts from six disciplines: cardiac surgery (*n* = 3), nursing (*n* = 4), clinical pharmacy (*n* = 2), rehabilitation medicine (*n* = 3), nutrition (*n* = 2), and psychology (*n* = 2). Experts were affiliated with eight tertiary hospitals and two universities in China. The mean years of professional experience was 18.6 years (range 11–32 years). All 16 experts participated in both Delphi rounds, and no expert withdrew during the process.

Two rounds of anonymous questionnaires were conducted from August to October 2023. The questionnaires contained open- and closed-ended questions covering the five stages of the DPS program (at admission, during hospitalization, before discharge, after discharge, and effect evaluation) and specific intervention measures. Experts rated the importance of each item on a 5-point Likert scale (1 = completely unimportant, 5 = extremely important) and provided written suggestions. Item scores were reported as mean ± SD. Round-specific response rates measured panel participation. Authority coefficient (Cr) was calculated as Cr = (Ca + Cs)/2 (16). After round one, mean, SD, CV, and qualitative feedback were summarized; disputed items were revised or merged for round two. In round two, experts reviewed the round-one summary and revision reasons, then re-rated the adjusted items. Consensus was predefined as mean importance ≥4.0 and coefficient of variation (CV) ≤ 0.25 (18). In addition, the Kendall W value was calculated to assess the degree of consensus among the expert panel regarding the importance ratings of all the items in the second round.

The team analyzed round-two data and identified consensus-reaching items. Crucial items not fully meeting the standards were finalized by incorporating experts' written opinions. In summary, The average importance score of all items was ≥4.0, and CV was ≤0.25. Second-round Kendall's W = 0.925 (*P* < 0.001), showing strong expert consensus. Response rate for both rounds was 100%. Second-round Ca = 0.923, Cs = 0.905, Cr = 0.914, demonstrating high expert authority. All consensus items were then integrated into the final structured “Systematic Discharge Plan for Patients after Heart Valve Replacement”, which was then implemented in clinical practice and evaluated in a retrospective cohort study.

### DPS nursing pathway

This study's DPS plan draws on 31 pieces of the best evidence summarized in the literature (13, 17). Led by a multidisciplinary team (MDT, including cardiac surgeons, specially trained nurses, and clinical pharmacists), this program covers the entire process from admission to 6 months after discharge and is implemented in five stages. Please refer to the following text for details. See Figure 1 and Table 1.

**Figure 1:**
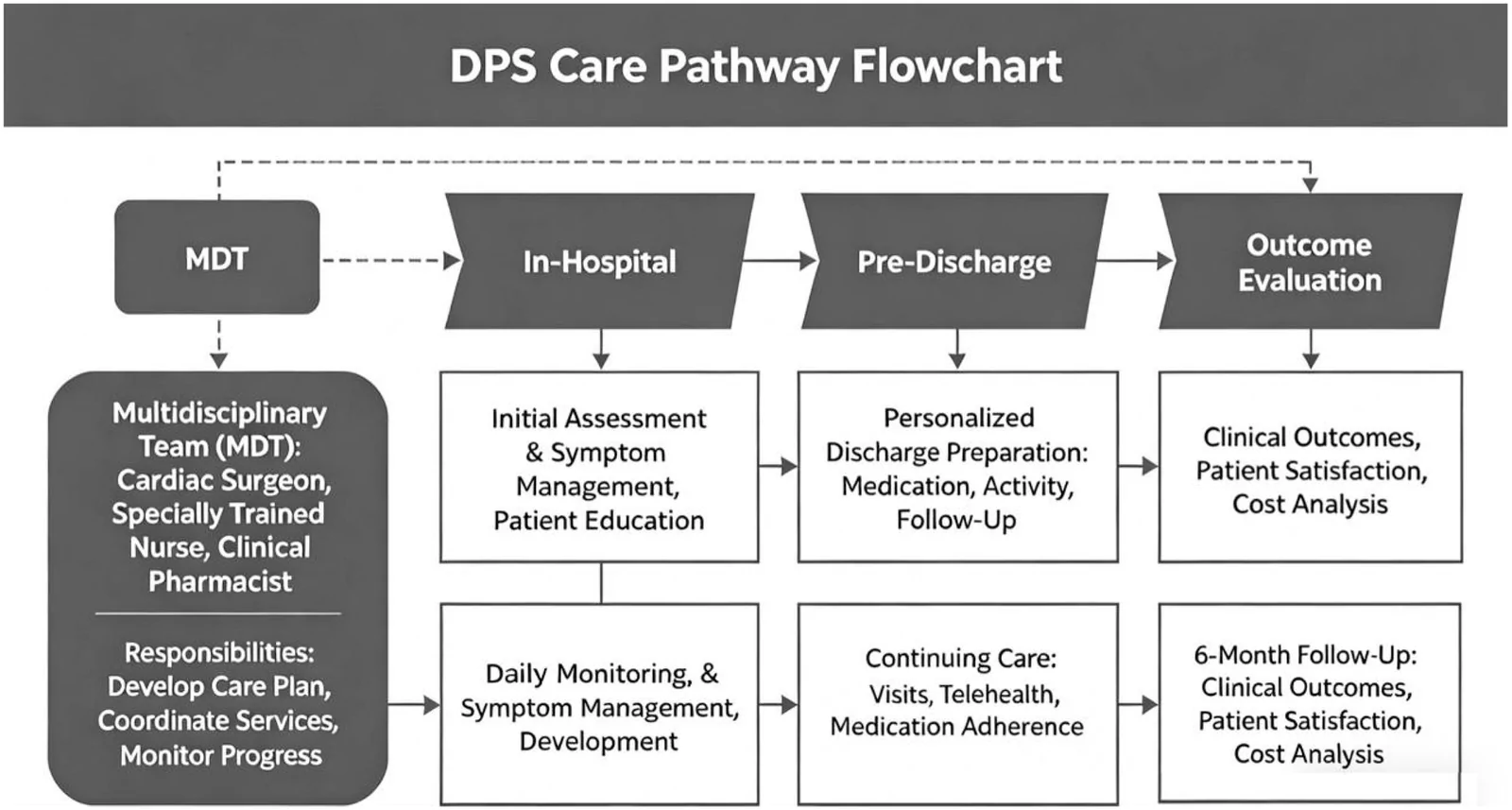
DPS nursing pathway flowchart.

**Table 1:** DPS nursing pathway for patients after heart valve replacement surgery.

Sections	Nursing measures	Responsible person	Tools/Methods	Importance scores	CV
Admission assessment	1. Conduct baseline assessment within 24 h of admission.	Specially trained nurse	Standardized admission assessment protocol	4.58 ± 0.58	0.127
	2. Evaluate the baseline level of discharge readiness.	All team members	Readiness for Hospital Discharge Scale (RHDS)	4.36 ± 0.62	0.142
	3. Assess disease cognition, social support, nutrition, psychology, fall risk, medication compliance, and rehabilitation needs.	Specially trained nurse	Modified Multidisciplinary Comprehensive Assessment Form	4.75 ± 0.52	0.109
	4. Jointly formulate personalized discharge goals with the patient and their family (short-term: master anticoagulation management skills; medium-term: resume daily activities one month after surgery; long-term: return to work and life six months after surgery).	All team members	King's Achievement Theory	4.17 ± 0.35	0.084
	5. Distribute rehabilitation manuals and play standardized educational videos.	All team members	Heart Valve Replacement Post-Operative Rehabilitation Manual; standardized health education video	4.62 ± 0.56	0.121
Plan development	1. Weekly core member case discussion to formulate individualized discharge preparation plan	All team members	Weekly MDT case discussion meeting	4.49 ± 0.61	0.136
	2. Temporary consultation with other experts when needed	MDT + other experts when needed	Temporary consultation mechanism	4.43 ± 0.41	0.093
	3. Share information via WeChat work group	All team members	WeChat work group	4.56 ± 0.49	0.107
	4. Conduct discharge assessment before discharge and provide personalized discharge guidance checklist	All team members	RHDS (required score ≥8); Quality of Discharge Teaching Scale (QDTS); discharge guidance checklist	4.76 ± 0.60	0.126
	5. Issue discharge summary and rehabilitation plan sheet	Nutritionist, nurses	Discharge summary template; rehabilitation plan sheet	4.63 ± 0.56	0.121
Teamwork	1. Cardiac surgeon: evaluate patient condition and surgical risks, formulate treatment plan, approve discharge plan, manage complications	Cardiac surgeon	Clinical evaluation and treatment planning	4.53 ± 0.59	0.130
	2. Specially trained nurse (including Discharge Coordination Specialist): coordinate plan, provide comprehensive health education, follow-up management, scale assessments, data recording, maintain WeChat group	Specially trained nurse (Discharge Coordination Specialist)	Care coordination, patient education, follow-up management, data tools	4.36 ± 0.50	0.115
	3. Clinical pharmacist: guide standardized anticoagulant management (warfarin), INR monitoring, management of adverse drug reactions	Clinical pharmacist	Anticoagulation management protocol, INR monitoring	4.44 ± 0.43	0.097
	4. Weekly MDT (surgeon + nurse + pharmacist) ward round to dynamically adjust rehabilitation plan	All team members	Weekly joint ward round	4.56 ± 0.52	0.114
	5. Temporary consultation with other experts when needed	All team members	Temporary consultation mechanism	4.51 ± 0.50	0.111
Patient education	1. One-on-one health education within 24-48 h post-surgery (anticoagulation, INR monitoring, wound care, complication identification)	Specially trained nurse	One-on-one education session	4.34 ± 0.46	0.106
	2. Weekly refresher training and Q&A assessment (mastery rate >90%)	Specially trained nurse	Refresher training and Q&A assessment	4.58 ± 0.46	0.100
	3. Clinical pharmacist guides proper anticoagulant use and self-monitoring of INR, assists in recording medication log	Clinical pharmacist	Medication log	4.38 ± 0.50	0.114
	4. Nurse guides cardiac rehabilitation exercises (ankle pump, deep breathing, bedside sitting, standing, assisted walking)	Specially trained nurse	Standardized rehabilitation protocol (day 1-6 stepwise guidance)	4.32 ± 0.53	0.123
	5. Nurse conducts nutritional screening, refers to nutritionist if needed	Specially trained nurse (refer to nutritionist if needed)	Nutritional screening form	4.43 ± 0.41	0.093
	6. SAS/SDS screening within 48 h post-surgery; routine psychological support for mild abnormalities, referral to psychologist for moderate/severe	Specially trained nurse (refer to psychologist if needed)	SAS/SDS scales	4.36 ± 0.52	0.119
	7. Nurse teaches identification of emergency situations (thromboembolism, bleeding, heart failure) and provides emergency contact card	Specially trained nurse	Emergency contact card	4.85 ± 0.69	0.142
	8. Weekly group health lectures and online Q&A via WeChat group (alternately by nurse and clinical pharmacist)	Specially trained nurse + clinical pharmacist (alternating)	WeChat group health lectures and Q&A	4.53 ± 0.34	0.075
Post-discharge follow-up	1. Within 1 week after discharge: first phone follow-up to assess rehabilitation status, medication compliance, complications	Specially trained nurse	Phone follow-up	4.38 ± 0.54	0.123
	2. At 1, 3, and 6 months after discharge: outpatient follow-ups by nurse, reassess self-management ability, SF-36, SAS/SDS, 6MWT, cardiac function (NYHA class)	Doctors, nurses	Outpatient follow-up with self-management scale, SF-36, SAS/SDS, 6-minute walk test, NYHA classification	4.54 ± 0.52	0.115
	3. Clinical pharmacist provides remote medication guidance based on INR results	All team members	Remote medication guidance (based on INR)	4.82 ± 0.61	0.127
	4. Assist in scheduling first outpatient follow-up appointment (1-2 weeks after discharge)	Specially trained nurse	Appointment scheduling service	4.62 ± 0.60	0.130
Emergency support	1. Nurse teaches patient and family to identify emergency situations (thromboembolism, bleeding, heart failure)	Specially trained nurse	Emergency situation education	4.36 ± 0.54	0.124
	2. Provide emergency contact card	All team members	Emergency contact card	4.39 ± 0.47	0.107
	3. Provide 24-hour consultation hotline	Specially trained nurse	24-hour consultation hotline	4.76 ± 0.52	0.109
	4. WeChat group for rapid response to emergency questions	All team members	WeChat group rapid response mechanism	4.49 ± 0.57	0.127

(1) Composition and Responsibilities of MDT.

The MDT includes: Cardiac Surgeon (1)–evaluates condition/surgical risks, makes treatment plan, approves discharge, manages complications. Specialized-trained Nurses (2, one as Discharge Coordination Specialist)–coordinate plan, provide health education, follow-up, assessments, data recording, maintain WeChat group. Clinical Pharmacist (1)–guides anticoagulation (warfarin), INR monitoring, adverse reaction management. The team holds weekly case discussions to develop individualized discharge plans and uses a WeChat group for communication.

(2) Systematic Discharge Preparation Service Plan.

#### Phase 1: at admission

Within 24 h, nurses conducted initial assessment using RHDS and a multidisciplinary form (disease awareness, social support, nutrition, psychology, fall risk, medication compliance, rehabilitation). Personalized discharge goals were set jointly with patients/families based on King's Achievement Theory (19) (short-term: anticoagulation skills; medium-term: daily activities at 1 month; long-term: return to work/life at 6 months). Goal attainment was reviewed at each follow-up contact, and the care plan was adjusted accordingly. Rehabilitation manuals and educational videos were provided.

#### Phase 2: during hospitalization

(a) Disease awareness education within 24–48 h post-surgery (anticoagulation, INR, wound care, complications), with weekly refresher and Q&A (target mastery >90%). (b) Medication management by clinical pharmacist (warfarin, INR self-monitoring, medication log). (c) Cardiac rehabilitation exercise (day 1–2: ankle pumps, deep breathing; day 3–5: sitting, standing, assisted walking); 6-minute walk test before discharge. (d) Nutritional screening and referral if at risk. (e) Psychological screening with SAS/SDS within 48 h post-surgery; routine support for mild issues, psychologist referral for moderate/severe. (f) Emergency guidance (thromboembolism, bleeding, heart failure) and emergency contact card. (g) Weekly MDT ward rounds and ad-hoc consultations.

#### Phase 3: before discharge (personalized preparation)

Nurse reassessed RHDS (≥8) and QDTS. Personalized discharge checklist provided. Pharmacist confirmed anticoagulation plan. First outpatient visit arranged (1–2 weeks after discharge). 24 h hotline and WeChat follow-up QR code provided. Discharge summary and rehabilitation plan issued.

#### Phase 4: after discharge (continuity of care)

Within 1 week: phone follow-up (rehabilitation, adherence, complications). At 3, 6 months: nurse-led outpatient follow-up (self-management, SF-36, SAS/SDS, 6MWT, NYHA class). Pharmacist provides remote INR-guided advice. Weekly WeChat group lectures and Q&A (alternating nurse/pharmacist).

#### Phase 5: effect evaluation (end-of-term assessment)

At discharge, 3 months, and 6 months: nurse assesses self-management ability, quality of life, psychological state, exercise tolerance, cardiac function, unplanned readmission rate, and satisfaction. Discharge readiness (RHDS) and discharge guidance quality (QDTS) are assessed at discharge only. Data reviewed by cardiac surgeon.

Intervention fidelity was monitored through weekly MDT records, follow-up logs, and WeChat group records. Overall adherence was high, with more than 90% completion for scheduled follow-up contacts, WeChat participation, education sessions, and remote INR-guided advice. Missing outcome data were handled using multiple imputation under the missing-at-random assumption.

### Research subjects

A retrospective consecutive selection was made of 323 patients who underwent heart valve replacement surgery at Tianjin Medical University Second Hospital from February 2023 to January 2025 and had complete follow-up data. Our hospital implemented DPS nursing starting from February 2024. To reduce inter-group selection bias, the nearest neighbor matching method in the propensity score matching (PSM) was adopted, and matching was performed at a 1:1 ratio. The specific steps are as follows: Based on baseline covariates such as age, gender, BMI, education level, ASA classification and surgical type, a logistic regression model is used to calculate the propensity score for each patient. The threshold value for the caliper was set as 0.2 times the standard deviation of the logit value of the propensity score, and non-replacement matching was used. The patients in the group before the implementation of the nursing plan (February 2023–January 2024) were matched with those in the group after the implementation (February 2024–January 2025). After matching, the balance of covariates between the two groups was evaluated using the standardized mean difference, and good balance was indicated by an absolute standardized mean difference (SMD) < 0.1 (see Figure 2).

**Figure 2:**
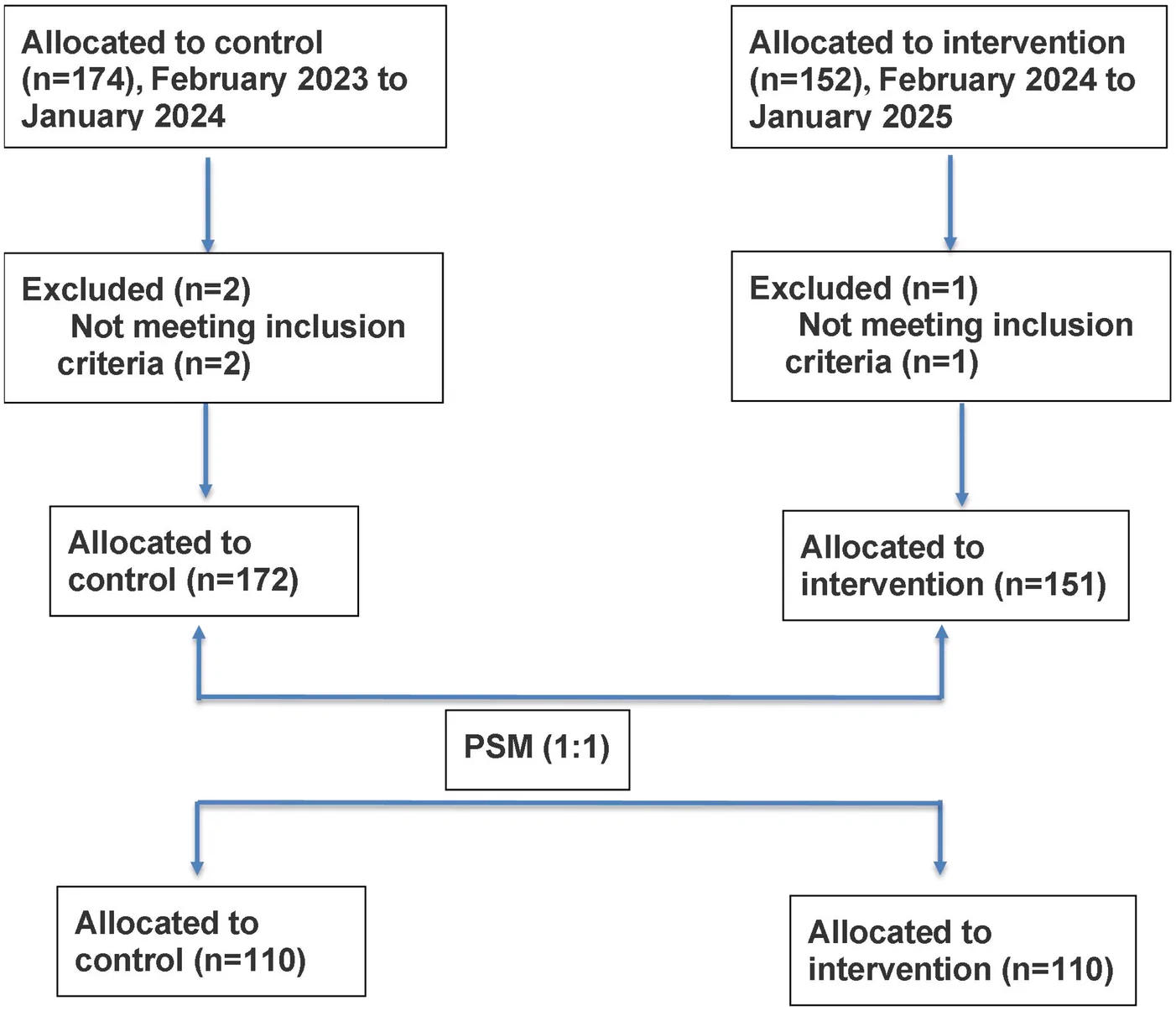
PSM process.

Finally, after the matching process was completed, patients who received conventional treatment from February 2023 to January 2024 (the control group, *n* = 110), and patients who received DPS treatment from February 2024 to January 2025 (the DPS group, *n* = 110). This study has been reviewed and approved by the Medical Ethics Committee of Tianjin Medical University Second Hospital [Ethical Number: KY2026K209].

This was a retrospective cohort study; the sample size was determined by the number of eligible patients admitted during the study period (February 2023–January 2025). A formal *a priori* sample size calculation was not performed. A *post hoc* power analysis showed that the matched sample of 110 patients per group provided 80% power at a two-sided *α* of 0.05 to detect a moderate between-group difference (Cohen's d = 0.5) in the primary outcomes.

### Inclusion and exclusion criteria

Inclusion Criteria: 1) Age ≥18 years; 2) Meeting the diagnostic criteria for valvular heart disease requiring heart valve replacement surgery as stipulated in the 2021 ESC/EACTS Guidelines for the Management of Valvular Heart Disease (20), and successfully undergoing heart valve replacement surgery; 3) Possessing basic communication, comprehension, and reading/writing skills; 4) Complete clinical data and follow-up information.

Exclusion Criteria: 1) Patients with severe adverse events during the operation; 2) Those undergoing two or more types of cardiac surgeries or major surgeries on other thoracic organs simultaneously; 3) Those with a history of brain diseases or neurological complications during the perioperative period; 4) Those unable to regain consciousness or having other causes of consciousness disorders after the operation; 5) Those with mental disorders or cognitive decline; 6) Those with incomplete clinical data, which affects the evaluation of the results; 7) Those lost to follow-up or having incomplete data.

In this study, all patients who underwent heart valve replacement surgery strictly followed the diagnostic criteria and surgical indications as stipulated in the 2021 ESC/EACTS Guidelines for the Management of Heart Valve Diseases (20). These were evaluated and confirmed by a multidisciplinary team consisting of cardiologists, cardiac surgeons, radiologists, and anesthesiologists. The specific inclusion criteria mainly included: being diagnosed with severe valvular heart disease requiring heart valve replacement surgery (including but not limited to severe aortic stenosis, mitral regurgitation). The patients were considered to have indications for heart valve replacement surgery and no absolute contraindications after comprehensive assessment by the cardiac team.

Termination criteria: These were pre-defined by the research team based on clinical guidelines and departmental protocols. Patients could be discharged when their vital signs were stable, they could tolerate oral diet, their level of self-care ability reached the predetermined grade, the incision healed well, and there were no serious complications requiring hospitalization.

### Nursing methods

Routine nursing: The control group received routine postoperative care and follow-up. The content included: During hospitalization, routine preoperative education was provided, postoperative condition observation was conducted, medication guidance was given, and basic rehabilitation suggestions were provided; at discharge, the responsible nurse provided routine discharge medication, activity, diet, and follow-up guidance; after discharge, the dedicated follow-up nurse conducted follow-up calls at 3 months and 6 months to understand the patient's condition and provide general guidance.

DPS nursing: The observation group received DPS nursing. The content is detailed in Figure 1 and Table 1.

### Data collection

Collected patient data: age, sex, BMI, hypertension, diabetes, smoking, drinking, home caregivers, and education level; clinical data: New York Heart Association (NYHA) cardiac function classification, Visual Analogue Scale (VAS) score, operation duration; outcome indicators: the time required to meet the discharge criteria, discharge readiness score, discharge guidance quality score; record the incidence of unplanned readmission within 6 months after surgery (Unplanned readmission was defined as any unplanned hospital admission within 6 months after discharge, including readmissions to our hospital or other hospitals, identified through medical records and telephone follow-up.) and the occurrence of complications during the disease course [pulmonary infection, new or progressive pulmonary infiltrates on chest imaging plus fever or leukocytosis requiring antibiotic treatment; arrhythmia, new-onset arrhythmia requiring pharmacological treatment, cardioversion, or prolonged monitoring; acute kidney injury, according to KDIGO criteria (increase in serum creatinine ≥0.3 mg/dL within 48 h or ≥1.5 times baseline within 7 days, or urine output <0.5 mL/kg/h for 6 h); incision infection, superficial or deep surgical site infection according to CDC criteria; low cardiac output syndrome, need for inotropic or vasopressor support or mechanical circulatory support due to cardiac index <2.0 L/min/m^2^; stroke, new focal neurological deficit confirmed by neuroimaging; delirium, diagnosed using the Confusion Assessment Method for the ICU (CAM-ICU) or DSM-5 criteria.); collect the self-management ability, quality of life, and nursing satisfaction scores at discharge and at 3 and 6 months after discharge.

### Standard for evaluation

1. Readiness for Discharge Scale (RHDS) (21, 22). This scale consists of four dimensions: personal status, common sense, coping ability, and expected support, with 21 items. Each item is scored 0–10, total 0–210. The Chinese version demonstrated good internal consistency (Cronbach's *α* = 0.97) and content validity (CVI = 0.97). An average item score ≥8 (total ≥168) was used as the cutoff for adequate discharge readiness.
2. Quality of Discharge Teaching Scale (QDTS) (23, 24). This consists of three dimensions: the content to be acquired, the actual acquired content, and the guidance skills and effects. It contains a total of 24 items. It is scored on a scale of 0–10, with a total score ranging from 0 to 240 (with the average score of each item ranging from 0 to 10). The Chinese version demonstrated good reliability (Cronbach's *α* = 0.924; split-half = 0.847) and content validity (CVI = 0.98). No formal cutoff; analyzed as a continuous variable. And evaluations were conducted before discharge.
3. Coronary Heart Disease Self-Management Ability Scale (CSMS) (25). This scale consists of seven dimensions: management of bad habits, general life management, symptom management, emergency management, disease knowledge acquisition management, treatment compliance management, and emotional cognition management. It contains a total of 27 items. The total score ranges from 27 to 135 points. The higher the score, the better the self-management behavior. The Chinese version demonstrated good internal consistency (Cronbach's *α* = 0.913; dimensions 0.818–0.881). No formal cutoff; analyzed as a continuous variable. Evaluations at discharge, 3 months, and 6 months post-discharge.
4. Chinese Cardiovascular Patient Quality of Life Assessment Questionnaire (CCQQ) (26). This scale includes dimensions such as physical condition, rehabilitation exercises, angina pectoris, palpitations, shortness of breath, impact of the disease on life, disease cognition, outlook on life and death, treatment satisfaction, satisfaction with medical staff, dietary situation, insomnia, self-perceived health status, sexual life status, recreational activities, depression of mood, anxiety of mood, memory, intelligence, confidence in life, relationship with relatives, work ability, and work interpersonal relationships, etc. There are a total of 24 items. The total score ranges from 0 to 154 points. The higher the score, the better the quality of life. This scale includes 24 items, total score 0–154. The Chinese version demonstrated acceptable reliability (Cronbach's *α* comparable to SF-36 *α* = 0.76). No formal cutoff; analyzed as a continuous variable. Evaluations at discharge, 3 months, and 6 months post-discharge.
5. Nursing Satisfaction Survey Form (NSNS) (27, 28). This scale covers dimensions such as the communication skills of nursing staff, professional competence, the timeliness and adequacy of health education, and the responsiveness to patients' needs. The total score ranges from 0 to 100, with a higher score indicating a higher level of patient satisfaction with the nursing services. The version demonstrated good reliability (Cronbach's *α* = 0.837). No formal cutoff; analyzed as a continuous variable. Evaluations at discharge, 3 months, and 6 months post-discharge.

### Statistical analysis

SPSS 27.0 (IBM, Armonk, NY, USA) was used for data analysis. The nearest neighbor matching method in PSM was adopted, and the matching was conducted at a 1:1 ratio. Based on the baseline covariates, a logistic regression model was used to calculate the propensity score for each patient. The threshold for the caliper value was set as 0.2 times the standard deviation of the logit of the propensity score, and non-replacement matching was employed. All subsequent data analyses were conducted using the data after PSM. Normality and homogeneity of variance tests were performed for all variables. For continuous variables that followed a normal distribution and had homogeneity of variance, the mean ± standard deviation ($\bar{x}\pm s$) was used for description, and t-tests were employed for comparisons between groups; for non-normal distribution of measurement data, the median and interquartile range were used for representation, and Wilcoxon tests were applied for comparisons between groups. Count data were expressed as frequencies and percentages, and *χ*2 tests were conducted. For the longitudinal outcomes (CSMS, CCQQ, and NSNS), linear mixed models were fitted using the lmerTest package in R (version 4.x) with the formula score ∼ time*group + (1|id), where time (discharge, 3 months, 6 months) and group (Control, DPS) were treated as fixed effects, and a random intercept for each patient was included. The scheme was constructed using the Delphi Method. All items meeting the predefined criteria (mean importance≥4.0, coefficient of variation ≤0.25) indicate that the construction was successful. *P* < 0.05 indicated statistically significant differences.

## Results

### Comparison of general information

After PSM, there were no differences between the two groups in basic information such as age, gender, BMI, hypertension, diabetes, smoking history, drinking history, home caregivers, educational level, NYH classification, VAS classification, and operation duration (*P* > 0.05, SMD < 0.1). See Table 2.

**Table 2:** Comparison of general information.

Index	Before PSM		*P*	SMD	After PSM		*P*	SMD
	Control group (*n* = 172)	DPS group (*n* = 151)			Control group (*n* = 110)	DPS group (*n* = 110)		
Gender/*n* (%)			0.136	0.051			0.785	0.056
Male	85 (49.42)	87 (57.62)			63 (57.27)	65 (59.09)		
Female	87 (50.58)	64 (42.38)			47 (42.73)	45 (40.91)		
Age (year, $\bar{x}\pm s$)	65.43 ± 6.32	69.64 ± 7.03	<0.001	0.631	64.22 ± 7.41	65.46 ± 6.62	0.186	0.036
BMI (kg/m^2^, $\bar{x}\pm s$)	23.26 ± 1.53	21.05 ± 1.96	<0.001	1.275	22.99 ± 1.89	23.16 ± 2.00	0.513	0.085
Highest education attained/*n* (%)			0.046	0.124			0.500	0.044
High school and above	89 (51.74)	69 (45.70)			56 (50.91)	51 (46.36)		
Junior high school and below	83 (48.26)	82 (54.30)			54 (49.09)	59 (53.64)		
Alcohol consumption history/*n* (%)			0.502	0.085			0.681	0.096
Yes	103 (59.88)	96 (63.58)			66 (60.00)	63 (57.27)		
No	69 (40.12)	55 (36.42)			44 (40.00)	47 (42.73)		
Smoking history/*n* (%)			0.154	0.097			0.342	0.063
Yes	86 (50.00)	63 (41.72)			52 (47.27)	45 (40.91)		
No	86 (50.00)	88 (58.28)			58 (52.73)	65 (59.09)		
Diabetes/*n* (%)			0.625	0.066			0.702	0.058
Yes	26 (15.12)	19 (12.58)			17 (15.45)	15 (13.64)		
No	146 (84.88)	132 (87.42)			93 (84.55)	95 (86.36)		
Hypertension/*n* (%)							0.603	0.089
Yes	36 (20.93)	25 (16.56)	0.584	0.071	22 (20.00)	19 (17.27)		
No	136 (79.07)	126 (83.44)			88 (80.00)	91 (82.73)		
Duration of the surgery (h, $\bar{x}\pm s$)	3.71 ± 0.34	3.97 ± 0.42	<0.001	0.693	3.84 ± 0.46	3.96 ± 0.47	0.067	0.054
ASA classification/*n* (%)			0.652	0.041			0.785	0.078
Ⅰ∼Ⅱ	99 (57.56)	81 (53.64)			64 (58.18)	62 (56.36)		
Ⅲ	73 (42.44)	70 (46.36)			46 (41.82)	48 (43.64)		
NYHA classification/*n* (%)			0.741	0.085			0.883	0.096
Ⅰ∼Ⅱ	51 (29.65)	40 (26.49)			33 (30.00)	32 (29.09)		
Ⅲ∼Ⅳ	121 (70.35)	111 (73.51)			77 (70.00)	78 (70.91)		
Home care/*n* (%)			0.613	0.071			0.762	0.059
Yes	128 (74.42)	105 (69.54)			81 (73.64)	79 (71.82)		
No	44 (25.58)	46 (30.46)			29 (26.36)	31 (28.18)		

### Comparison of RHDS scores

Compared with the Control group, the total score of RHDS and the scores of the four dimensions of personal status, common sense, coping ability, and expected support in the DPS group were significantly increased (*P* < 0.05). See Table 3.

**Table 3:** Comparison of RHDS scores.

Index	Control group (*n* = 110)	DPS group (*n* = 110)	*t/Z*	*P*
Personal status [score, M (Q1, Q3)]	35.00 (31.00, 39.75)	41.00 (36.75, 44.25)	−5.996	<0.001
Common sense (score, $\bar{x}\pm s$)	40.35 ± 7.78	52.82 ± 6.72	−12.806	<0.001
Coping ability (score, $\bar{x}\pm s$)	21.09 ± 3.33	26.65 ± 3.03	−13.034	<0.001
Expected support [score, M (Q1, Q3)]	29.00 (28.00, 31.00)	37.00 (35.00, 39.00)	−11.957	<0.001
Total score (score, $\bar{x}\pm s$)	126.68 ± 12.03	157.14 ± 9.14	−21.243	<0.001

### Comparison of QDTS scores

Compared with the Control group, the total score of QDTS, the scores of the three dimensions of the content to be obtained, the actual acquired content, and the guidance skills and effects in the DPS group were all significantly increased (*P* < 0.05). See Table 4.

**Table 4:** Comparison of QDTS scores.

Index	Control group (*n* = 110)	DPS group (*n* = 110)	*t/Z*	*P*
Content to be acquired [score, M (Q1, Q3)]	55.00 (53.00, 57.00)	56.00 (54.00, 58.00)	−2.450	0.014
Actual acquired content [score, M (Q1, Q3)]	37.00 (35.25, 39.00)	45.00 (44.00, 46.00)	−12.713	<0.001
Guidance skills and effects (score, $\bar{x}\pm s$)	94.50 ± 4.51	103.03 ± 5.29	−20.199	<0.001
Total score (score, $\bar{x}\pm s$)	186.84 ± 6.11	203.95 ± 6.56	−13.002	<0.001

### Comparison of follow-up data

Compared with the Control group, the scores of CSMS, CCQQ and NSNS in the DPS group significantly increased at 3 months and 6 months (*P* < 0.05), there was no statistically significant difference at discharge (*P* > 0.05). Compared with the 3-month scores of the same group, the scores of CSMS, CCQQ and NSNS of the two groups at 6 months also significantly increased (*P* < 0.05). See Table 5. Linear mixed models further showed significant time × group interactions for CSMS, CCQQ, and NSNS [CSMS: F(2,436) = 77.73; CCQQ: F(2,436) = 93.92; NSNS: F(2,436) = 67.53; all *P* < 0.001; Table 6], indicating that the DPS group had a steeper improvement trajectory over the 6-month follow-up, with between-group differences widening from discharge to 6 months.

**Table 5:** Comparison of follow-up data.

Index	Time	Control group (*n* = 110)	DPS group (*n* = 110)	*t*	*P*
CSMS (score, $\bar{x}\pm s$)	At discharge	67.52 ± 9.26	68.23 ± 8.54	−0.591	0.555
	3 months later	70.34 ± 5.57	77.58 ± 6.15	−9.242	<0.001
	6 months later	103.22 ± 7.10**	121.16 ± 7.18**	−18.805	<0.001
CCQQ (score, $\bar{x}\pm s$)	At discharge	74.63 ± 7.39	75.71 ± 8.05	−1.037	0.301
	3 months later	80.82 ± 4.78	89.72 ± 5.98	−12.271	<0.001
	6 months later	114.28 ± 10.77**	132.97 ± 11.18**	−12.741	<0.001
NSNS (score, $\bar{x}\pm s$)	At discharge	58.32 ± 8.26	59.47 ± 8.53	−1.016	0.331
	3 months later	64.66 ± 4.91	73.05 ± 4.70	−13.051	<0.001
	6 months later	77.81 ± 5.34**	89.36 ± 6.04**	−15.189	<0.001

Compared with the same group at 3 months.

** *P* < 0.001.

**Table 6:** Time × group interaction effects from linear mixed models for CSMS, CCQQ, and NSNS.

Outcome	Parameter	Estimate	SE	95% CI	*t*	*P*
**CSMS**	Group × Time: DPS at 3m	0.53	1.017	−1.47–2.53	0.52	0.604
	Group × Time: DPS at 6m	11.23	1.017	9.23–13.23	11.04	<0.001
**CCQQ**	Group × Time: DPS at 3m	5.82	1.151	3.57–8.07	5.06	<0.001
	Group × Time: DPS at 6m	15.61	1.151	13.36–17.86	13.56	<0.001
**NSNS**	Group × Time: DPS at 3m	7.24	0.918	5.44–9.04	7.89	<0.001
	Group × Time: DPS at 6m	10.4	0.918	8.60–12.20	11.33	<0.001

Overall time × group interaction (Type III *F* test): CSMS: F(2, 436) = 77.73, *P* < 0.001; CCQQ: F(2, 436) = 93.92, *P* < 0.001; NSNS: F(2, 436) = 67.53, *P* < 0.001. Models: score∼time*group + (1|id) fitted with REML. Time = discharge (reference), 3 months, 6 months; Group = Control (reference), DPS. The interaction term “Group × Time: DPS at 3m” represents the additional change in the DPS group at 3 months relative to the change in the Control group over the same period; positive values indicate greater improvement in the DPS group.

### Compare the rates of complications and unplanned readmissions within six months

The unplanned readmission cases in the Control group were 17 (15.45), while in the DPS group it was 7 (6.36). The unplanned readmission rate in the DPS group was significantly lower than that in the Control group (*P* < 0.05). Regarding complications, the rates in the Control group for pulmonary infection, arrhythmia, acute kidney injury, incision infection, hypoperfusion syndrome, stroke, delirium were 14 (12.73), 11 (10.00), 12 (10.91), 4 (3.64), 5 (4.55), 2 (1.82), 17 (15.45) respectively, while those in the DPS group were 5 (4.55), 5 (4.55), 6 (5.45), 2 (1.82), 3 (2.73), 1 (0.91), 6 (5.45) respectively. Among them, the rates of pulmonary infection, delirium, and the total complication rate in the DPS group were significantly lower than those in the Control group (*P* < 0.05). See Table 7.

**Table 7:** Compare the rates of complications and unplanned readmissions within six months.

Index		Control group (*n* = 110)	DPS group (*n* = 110)	$\chi^{2}$	*P*
Unplanned readmission/n (%)		17 (15.45)	7 (6.36)	4.677	0.031
Complication/n (%)	Pulmonary infection	14 (12.73)	5 (4.55)	4.666	0.030
	Arrhythmia	11 (10.00)	5 (4.55)	2.426	0.119
	Acute kidney injury	12 (10.91)	6 (5.45)	2.178	0.140
	Incision infection	4 (3.64)	2 (1.82)	-	-
	Hypoperfusion syndrome	5 (4.55)	3 (2.73)	-	-
	Stroke	2 (1.82)	1 (0.91)	-	-
	Delirium	17 (15.45)	6 (5.45)	5.875	0.015
	Total	65 (59.09)	28 (25.45)	25.500	<0.001

## Discussion

DPS aims to enhance patients' self-management ability and safety compliance during the transition from the hospital to home through systematic health education, transitional coordination, and follow-up support. It has been regarded as an effective intervention strategy for reducing postoperative complications and unplanned readmission rates (29). Although existing evidence-based research supports the positive value of transitional care, more high-quality empirical studies are needed to verify the effectiveness of structured DPS intervention for surgical postoperative patients (30). The conclusion of this study indicates that compared with conventional care, structured discharge preparation services can significantly improve patients' CSMS, CCQQ, and NSNS after discharge, while effectively reducing the rates of unplanned readmission and the occurrence of complications such as pulmonary infection and delirium, suggesting that DPS may have clinical value in facilitating the smooth transition of postoperative patients and improving clinical outcomes.

Previous systematic reviews of Delphi studies have found that the definitions of consensus vary greatly and are insufficiently reported. Approximately 98% of the studies claimed to have evaluated consensus, but only 42% set a threshold in advance (31); In the improved Delphi method, the consistency and reliability of expert opinions are the core for evaluating the quality of the research. The results of this study indicate that all items meet the preset consensus standards (average score ≥4.0, CV ≤ 0.25), and the Kendall W value in the second round is 0.925 (*P* < 0.001), showing that the expert group has a very strong and statistically significant consistency in the importance they assign to each item of the DPS plan. This study not only clearly set the consensus standards in advance, but also strictly followed the predefined analytical framework, further enhancing the robustness of the research results. Moreover, the 100% response rate of the two rounds of questionnaires reflects the extremely high participation enthusiasm of the expert group. Veugelers et al. (32) pointed out that a high response rate is an important guarantee for the quality of the Delphi study, and is superior to the 80%–90% positive coefficient usually reported in previous similar studies. In this study, the expert authority coefficient Cr reached 0.914, which was higher than the common range of 0.80–0.90 reported in most nursing Delphi studies, reflecting that the expert group has accumulated profound academic attainments and rich practical experience in the fields of perioperative care in cardiac surgery, anticoagulation management, and rehabilitation medicine. In summary, these data not only jointly constitute the high-quality methodological basis for the construction of the DPS scheme, but also significantly enhance the clinical application value and promotion prospects of the constructed scheme. It is important to emphasize that the high Kendall's W and expert authority coefficient reflect strong agreement among the Delphi panel regarding the content and structure of the DPS plan, but they do not in themselves constitute evidence of clinical effectiveness. The observed improvements in clinical outcomes should be attributed to the implementation of the multifaceted DPS pathway as a whole. Given that the pathway comprises multiple interacting components delivered concurrently, we are unable to isolate the independent contribution of each element. However, we speculate that the combination of structured education, continuous follow-up, and multidisciplinary coordination may have played a synergistic role in enhancing patients' transitional experience and reducing adverse events.

The Readiness for Hospital Discharge refers to the patient's subjective perception of whether they are ready to leave the medical institution. It is an important indicator for predicting the patient's outcome after discharge (33). The results of this study showed that compared with the Control group, the total score of RHDS and the scores of the four dimensions of personal status, common sense, coping ability, and expected support in the DPS group were significantly increased (*P* < 0.05), suggesting that systematic and multidisciplinary discharge preparation services may be associated with the discharge readiness level of postoperative patients. Previous studies have shown that pre-discharge assessment, health education and discharge planning, post-discharge telephone follow-up, and caregiver participation are the most common components of transitional care intervention, and these interventions generally show positive effects in reducing readmission rates and improving patient satisfaction (34). In this study, the significant improvement in the personal status and coping ability dimensions of the DPS group is consistent with the conclusion in the above literature that transitional care intervention can enhance patients' self-perceived discharge readiness. It indicates that a structured discharge preparation service oriented towards patient needs can more comprehensively cover the physiological, psychological and knowledge reserve needs of patients.

The quality of discharge guidance is a key indicator for evaluating the effectiveness of the health education content and methods provided by nursing staff before patients are discharged (35). The results of this study showed that compared with the Control group, the total score of QDTS and the scores of the three dimensions of “to be acquired content”, “actual acquired content”, and “guidance skills and effects” in the DPS group have significantly increased (*P* < 0.05), suggesting that systematic discharge preparation services may contribute to higher-quality discharge guidance. This finding is consistent with previous research results. In the original study on the discharge readiness of adult medical and surgical patients, there was a significant positive correlation between the QDTS and RHDS, and high-quality discharge guidance can significantly improve the discharge readiness level of patients (23). The above evidence indicates that the quality of discharge guidance is a core antecedent variable influencing patients' discharge readiness. In this study, the comprehensive improvement in the three dimensions of the DPS group indicates that the discharge preparation service model led by nurses and with multidisciplinary collaboration can more accurately identify patients' discharge guidance needs and adopt more effective guidance methods to convey knowledge to patients, thereby achieving an overall improvement in the quality of discharge guidance.

The self-management ability after discharge is a key indicator for measuring the smooth transition of patients from the hospital to their home (36). This study used CSMS, CCQQ, and NSNS to evaluate the patients' daily self-management behaviors, perception of chronic disease care quality, and the degree of satisfaction with post-discharge nursing needs, in order to comprehensively assess their self-management ability and transition experience after discharge (37). The results of this study showed that compared with the Control group, the DPS group had significantly higher CSMS, CCQQ, and NSNS scores at 3 months and 6 months (*P* < 0.05). In addition, compared with the same group at 3 months, the CSMS, CCQQ, and NSNS scores of both groups at 6 months also significantly increased (*P* < 0.05), suggesting that the self-management ability after discharge showed a continuous improvement trend over time, and the positive effect of discharge preparation services was sustained throughout the follow-up period. The significant time × group interactions further indicated that DPS was associated with a steeper improvement trajectory in CSMS, CCQQ, and NSNS over the 6-month follow-up, rather than only with differences at isolated time points. This trajectory-level benefit supports the cumulative value of structured, continuous transitional care after discharge. This result is consistent with the existing evidence-based evidence. The intervention study based on the chronic disease self-management model pointed out that an intervention model led by nurses and based on caregivers, with continuous intervention at multiple time points during the perioperative period and after discharge, can effectively improve the self-management ability and quality of life of cancer surgery patients (36). Additionally, a study on patients after coronary artery bypass grafting surgery showed that planned discharge guidance can effectively enhance patients' knowledge level and self-efficacy, and there is a significant positive correlation between knowledge level and self-efficacy (38). The significant improvement in CSMS and CCQQ scores of the DPS group in this study, suggesting that the structured discharge preparation service, through systematic health education and transitional support, was associated with improved self-management ability. At the same time, the scores of both groups showed an upward trend over time during the follow-up period, indicating that postoperative patients will gradually adapt to home-based rehabilitation life over time, regardless of whether they received discharge preparation services, but the DPS group was at a higher level at each time point after the intervention, reflecting the cumulative effect of the structured intervention.

Unplanned readmission rate is a key metric for medical quality and transitional care effectiveness (39). The results of this study show that there were 17 unplanned readmissions in the Control group (15.45%), and 7 in the DPS group (6.36%). The unplanned readmission rate in the DPS group was significantly lower than that in the Control group (*P* < 0.05). In terms of complications, the incidence of pulmonary infection, delirium, and total complications in the DPS group was significantly lower than that in the Control group (*P* < 0.05). This finding is highly consistent with the conclusions of several high-quality studies in the current international context. Regarding the impact of discharge planning on the readmission rate, Aguilera's meta-analysis included 16 studies, and the meta-analysis results showed that TCPs were significantly associated with a reduced readmission rate (OR: 0.68, *P* < 0.0001), and were also associated with a shorter hospital stay (mean difference: −0.57, *P* < 0.00001) and a lower emergency visit rate (OR: 0.33, *P* < 0.0001) (40). Additionally, Collet's meta-analysis on multidisciplinary transitional care intervention included 49 trials involving 25,566 patients, and the results showed that MTCIs could reduce the readmission rate (RR: 0.88, 95% CI: 0.80–0.96) (41). In terms of complication prevention, Gillespie et al. (42). found that discharge education intervention could reduce the 30-day surgical site infection risk (OR = 0.84, 95% CI: 0.39–1.82) and possibly reduce the readmission rate (OR = 0.88, 95% CI: 0.56–1.38). The multidisciplinary collaborative research of the Kansas University Cardiac Surgery Readmission Committee also reported a similar trend. The interdisciplinary collaboration and pre-discharge checklist strategy reduced the cardiac surgery readmission rate from 10.9% to 9.6%, and the isolated CABG readmission rate from 10.2% to 6.1% (43). These results have significant clinical significance, suggesting that structured discharge preparation services may be associated with lower unplanned readmission and complication rates in postoperative patients, and may contribute to more efficient utilization of medical resources, alleviate the burden of family care, and provide effective nursing intervention strategies for promoting the safe and smooth transition of patients from the hospital to home.

In conclusion, this study successfully constructed a systematic DPS (Discharge Preparation Service) scheme through the modified Delphi method. All items achieved a high level of expert consensus (average importance ≥4.0, coefficient of variation ≤0.25). After controlling for confounding factors using PSM, compared with the conventional care group, the DPS group showed advantages in terms of discharge readiness, quality of discharge guidance, self-management ability at 3 months and 6 months after discharge, quality of life, and nursing satisfaction. Moreover, it also demonstrated advantages in terms of unplanned readmission rate and complication occurrence rate. This indicates that a patient-centered, structured multi-disciplinary collaborative discharge preparation service were associated with better discharge readiness, improved quality of discharge guidance, and enhanced self-management ability, and may have potential benefits in reducing the risk of short-term readmission. This scheme provides practical basis and reference for clinical promotion.

However, this study still has limitations. Firstly, its before-and-after design introduces potential temporal confounding. Although propensity score matching balanced measured baseline covariates, it cannot eliminate confounding from unmeasured factors or secular trends in clinical practice. Therefore, the observed differences should be interpreted as associations rather than causal effects. Secondly, it is a single-center study, which limits its external validity. Future multi-center and large-sample studies are needed to verify the universality of the model. Thirdly, the follow-up period is only 6 months, lacking long-term outcome data. Future studies should extend the follow-up period to more than 12 months and include long-term quality of life and functional recovery indicators. Fourthly, outcome assessment is mainly based on patient self-report, which may be subject to recall and social expectation biases. Moreover, a cost-benefit analysis has not been conducted. In the future, objective monitoring indicators (such as wearable devices) will be introduced and a health economics evaluation will be carried out to comprehensively assess the clinical value and economic feasibility of this model. Future studies should examine the role of complexity of care in shaping patients' readiness for discharge and their response to structured discharge services, considering that nursing-generated measures of complexity may help identify different care needs, resource requirements, and risks of adverse outcomes across patient populations (44–46).

## Data Availability

The raw data supporting the conclusions of this article will be made available by the authors, without undue reservation.
